# The tidal waves of connected health devices with healthcare applications: consequences on privacy and care management in European healthcare systems

**DOI:** 10.1186/s12911-017-0408-6

**Published:** 2017-01-17

**Authors:** Francois-André Allaert, Noël-Jean Mazen, Louis Legrand, Catherine Quantin

**Affiliations:** 1 Medical Evaluation Chair ESC and CEN Biotech, Dijon, Bourgogne France; 2 Bourgogne Franche-Comté University, Dijon, France; 3 Biostatistics and Bioinformatics (DIM), University Hospital, Dijon, France; Bourgogne Franche-Comté University, Dijon, France; 4 CNRS UMR 6306, Le2i, Bourgogne Franche-Comté University, F-21000 Dijon, France; 5 INSERM, CIC 1432, Dijon, France; Dijon University Hospital, Clinical Investigation Center, clinical epidemiology/clinical trials unit, Dijon, France; 6 Biostatistics, Biomathematics, Pharmacoepidemiology and Infectious Diseases (B2PHI), INSERM, UVSQ, Institut Pasteur, Université Paris-Saclay, F-21000 Paris, France

**Keywords:** Connected health devices, Healthcare applications, Privacy, Protection, Security, Societal impact

## Abstract

**Background:**

The market for Connected Health Devices (CHD) with healthcare applications is growing fast and should be worth several billion euros in turnover in the coming years. Their development will completely transform the organisation of our healthcare system, profoundly change the way patients are managed and revolutionizes disease prevention.

**Main body:**

The CHD with healthcare applications is a tidal wave that has societal impact calling into question the privacy of patients’ personal and healthcare information and its protection in secure systems. Rather than trying to stop the use of CHD, we must channel the wave by clearly examining the advantages versus the risks and threats to the patients, and find counter-measures for implementation. The main difficulty is channeling the wave in a way that is acceptable to CHD developers who otherwise will bypass the rules, even if they can be sued for it. Therefore, it appears necessary to implement guidelines that can be used by all developers, defining the minimum requirement for assuring the security of patient privacy and healthcare management.

**Conclusion:**

In European Healthcare Systems, there is an imperative need for establishing security guidelines that CHD producers could use to ensure compliance, so that patient privacy and healthcare management is safeguarded. The aim would be to implement the guidelines a posteriori rather than a priori control so as not to hamper innovation.

## Background

The evaluation of Connected Health Devices (CHD) raises questions with regard to both public health and the protection of individual privacy. The answers to these questions will, without doubt, have an impact on the industrial and economic success of the development of CHD. During a scientific symposium, held in France at the end of 2015, open to the general public, more than 500 participants including professionals from the pharmaceutical industry, the information technology industry and the healthcare system debated the need for a technological and ethical validation of CHD. These debates were chaired by the Vice President of the National Medical Council, and the Director of Public Relations and Research of the national commission for data protection (CNIL). This dual appraisal, involving society at large and the professional world, brought to light and pinpointed not only the immense potential of CHD, but also the obstacles to their development and solutions that could be implemented. This article addresses the potential security issues associated with CHD, which must be explored with regard to the efficacy of the health service provided and to the protection of individual privacy.

## A tidal wave that could turn our healthcare system upside down

The global market for CHD with healthcare applications is growing fast and should be worth several billion euros in turnover in the coming years. Many economists believe that their arrival will have a similar impact to that we experienced with the development of the Internet. Today, there are more than 97,000 downloadable healthcare applications, and hundreds of connected devices are already on the market [1]. Most of the currently available CHD with healthcare applications do not constitute major advances in themselves and can be considered gadgets rather than medical devices. Nonetheless, some of them already offer healthcare coaching services and advice, which may be good or bad; it may impact on the users’ well-being or their health. Similarities can be drawn, more or less, to the way in which the Internet has affected patients experience and knowledge in the face of serious illnesses [2]. Other applications, via more invasive measurements, for example, through the analysis of physiological cardiovascular parameters, are moving into a real medical field. For example, providing your doctor with information on your blood pressure [3] from your application could lead to a reaction from him/her if you have uncontrolled hypertension. One could even imagine automatic regulation, directly linked to blood pressure measurements, as is already the case for insulin pumps [4, 5]. It is important to underline that even the least sophisticated systems, like a step counter [6], could eventually be diverted from its original purpose in particular via certain information transmission processes that transfer data to a server. It is also easy to imagine that private health insurance companies may encourage clients to buy CHD with their healthcare applications and accept the transmission of information reflecting the clients’ healthy lifestyle, in exchange for gifts or bonuses for the «good» clients, and eventually higher insurance premiums for the more negligent clients. There is a precedent for this in the field of continuous positive pressure apparatus used in the management of patients with sleep apnoea syndrome. The apparatus is equipped with a chip that records the duration of its use. For patients who do not use the apparatus sufficiently, it may be taken away or the cost may no longer be covered by the health insurance agency. Following a complaint by certain patients’ associations, the French Council of State declared this measure illegal in 2014, not because of the principle, but simply because of legal considerations. They indicated that this measure could not be based on a decision of the health insurance agency alone but required a law, a law which may one day come into being. In the field of road transport, there are also systems that analyse the behaviour of drivers, and insurers reward those with a ‘less aggressive’ approach to driving.

It is clear that, for the first time, it is possible to evaluate the everyday behaviour of individuals using objective criteria via connected devices. The use of such CHD will increase and will impact every aspect of our lives, from smoking and the consumption of alcohol or psychotropic substances to compliance with treatments, or with physical activity and dietary guidelines [7]. Will CHD with healthcare applications eventually undermine our principle of national healthcare solidarity and will they lead to payment systems that vary depending on a person’s risk profile? This is a major societal issue. However, the possibility offered to people to pay for extra services in the field of health must not be restricted and on the contrary their widespread use may contribute to decrease their price and make them more affordable to everybody.

Beyond the potential effects of CHD with healthcare applications on healthcare management, they may also affect the organisation of the whole healthcare system. It is easy to imagine that in the very near future, systems that transmit a person’s physiological parameters will multiply; in a healthcare system that already has a shortage of doctors. The doctors will have to cope with an incessant flow of data to their practices; to avoid saturation, expert systems need to be set up at the level of the servers to reproduce the medical rationale and to provide patients with appropriate “medical” advice. This may be perfectly possible if such artificial intelligence systems are properly validated and made secure [8–10]. This new organisation of healthcare would facilitate the life of patients as well as that of doctors. However, the limits of such re-organisation healthcare systems need to be explored. Eventually, many patients could be followed in this way, which would deprive the medical community of part of its activities. This raises the major question as to who will control these information systems, which will play a substantial role in healthcare. What will happen if companies that manage these systems suddenly go bankrupt or if they decide to increase fees to unbearable levels? One could also fear that they may refuse to communicate information for a person to another system, thus forcing the user to remain a “captive” of the system that he/she initially subscribed to. The competition laws and the freedom of choice for individuals could be severely compromised. In addition, what would be the consequences if an ill-intentioned person hacked the system or if the system was based in a country with which economic or diplomatic tensions occurred? This would without doubt raise questions about the control, at least in Europe, of organisations likely to manage healthcare information systems. Going further on the way of a security policy analysis, we must also take into account the “worse scenario”: an event which has “obviously” a low probability of occurring but whose consequences could be disastrous. For example, today some companies selling cars are developing connected devices which measure your alcohol level through breath-analysers and may prevent you to start the car if you have drunk too much. This could be effectively a good thing to reduce car accidents. Imagine that a new law on prohibition appears like between 1920 to 1933 in the US and that the connected breath-analysers already exist, people could have asked to have it permanently connected to know if they are consuming alcohol. Imagine what could have been the possibility offered to the Nazis to track people in 1939 through the database if they were already available. They could have prosecuted them or send them to reeducation camps! Things who may appear good to us today may become a nightmare tomorrow if we do not take measures to prevent the risk of function creep. Some authors [11] have also imagined that this could frighten our democracy through the vote of the citizens.

Another challenge in the development of these CHD with healthcare applications is maintaining equal access to care for all. Many of these systems would provide an aid to prevention, to diagnosis and to the therapeutic follow-up of patients. However, one may fear that the investments necessary to set up such systems would make their products relatively expensive. Given their proven usefulness, they may need to be managed by a government agency or by private or mutual insurance systems. This could give rise to the appearance of a two-speed e-medicine system, in which those who can afford it will benefit from permanent follow-up, whereas the others will have to make do with periodic consultations.

Finally, the CHD with healthcare applications may be a way to make the young generations aware, for the first time and directly, of prevention. This, indeed, underlines one of the major weaknesses of the way prevention is organised today, that is to say perceived as necessary only by persons for whom the risk has become major. In fact, measures to have a healthier lifestyle should be followed from the earliest ages. Progress has certainly been made, notably in healthy eating and sports activities, thanks to repeated messages in the media, but the effort made and the cost have been immense compared with the results [12], without taking into account the fact that it takes years to affect the behaviour of our fellow citizens. Communicating prevention messages via tools to which the young generations are particularly attached, and using game-type strategies, could have a positive effect and at last reach a target that until now has been impervious to prevention messages.

Many other examples can be found to illustrate to what extent these CHD and healthcare applications will turn the organisation of our healthcare system upside down, and yet, we have only tackled a minor part of what will be available tomorrow, given the extremely rapid technological progress [13–15].

## Channel the wave rather than try to stop it

Given the potential of this tidal wave of CHD with healthcare applications, where vested economic interests are involved, it is unlikely to disappear and it is pointless trying to stop it. Instead, it isfar better to channel it, in order to generate a positive impact for patients, both individually and at the level of the healthcare system as a whole.

One of the key questions concerns the assessment of technological and medical validation of CHD with healthcare applications for their value as a veritable healthcare service. Of course, in many fields, the issues are not major and the impact will not be particularly important. For example, if a system that counts the number of steps per day does not perform very well, it will be a matter of deceit on the product’s quality, which from a legal point of view is unacceptable. However, the issues are of another and more serious dimension, if we consider a CHD that measures the aqueous humour of the eye, then determines glycaemia and thus controls an insulin pump. It is therefore important, in every medical condition, to evaluate CHD according to objective criteria.

Concerning the methodology, when such products claim to provide a health service, they must be considered, according to the European Council Directive 93/42/EEC of 14 June 1993 concerning medical devices. They coveran extremely vast category of products comprising different classes (type I to IV) [of devices ranging from sticking plasters to hip prostheses and from compression stockings to pacemakers. A study of the reliability and quality of measurements taken by a CHD is essential. As it is often the case with evaluation of medical devices, such studies should by randomized clinical trials, or even observational studies conducted in everyday practice, since even the notion of a comparator or a blinded trial is difficult to imagine. The evaluation will not give rise to hostile reactions from the designers or producers of the CHD with healthcare applications, because what will be required of them will be “fair” and proportional to medical safety concerns. The only misgivings of the companies present at the aforementioned debate concerned not the principle, but the slow pace of the regulatory procedures that govern clinical research and today lead to delays of 6 months or more. Without doubt, observational studies would be better suited than randomized, controlled, double-blind clinical research versus a comparator. It will be on the basis of these studies that reimbursements for the CHD with healthcare applications, considered medical devices, could be requested, provided that the brand name of the device is listed as one of the products or services reimbursed by the health insurance agency.

The second question concerns the respect for and protection of individual privacy with regard to the information collected and processed by these devices. The framework for this evaluation is the law 78–17 of the 6th January, 1978, relative to information technologies, to computer files and to privacy articles modified numerous times, notably to incorporate the European directive 95/46/EC of the 24^th^ October, 1995 relative to the protection of personal data into French law. This text states that any processing of personal information must be declared and that any processing of personal healthcare data must be authorized beforehand. These procedures aim to ensure that the systems provide the necessary protection not only the confidentiality of data, but also their integrity and their availability. These three notions (confidentiality, integrity and availability) constitute the pillars of data security, and thus underline that confidentiality is not the only concern. Confidentiality is of course an extremely fundamental notion as it has a direct impact on the private life of patients, and implies that only those authorized should have access to this information. Access to this information by a person without authorization would be in violation of professional secrecy as defined by the articles 226–13 and 14 of the French penal code with regard to health data. The transmission of files to an unauthorized party would be regarded as diverting the file from its intended purpose, which is punishable in article 226–16 of the penal code by 5 years of imprisonment and a fine of 300,000 euros. In the same way, the person responsible for the security of computer system must implement the necessary measures to ensure that data are not damaged, or deformed, or destroyed. Finally, and without prejudice, other relatively restrictive measures, the fundamental rights of patients must be respected and notably their right to be informed, their right of access to their information, to rectify or to transfer their information, and their right to “be forgotten” which means they have the right to have information concerning them erased. It is perfectly possible to meet all of these different constraints, but as soon as health data are involved, the procedures to request authorization are particularly long.

Companies consider this element particularly prejudicial because these constraints indirectly lead to a distortion of competition with regard to CHD with healthcare applications. Indeed, the producers of the devices are located in various countries, in particular the United States or Asian countries, which do not have the same level of protection of individual information privacy. Of course, in law, all products available in the European Union are subject to the same obligations with regard to the protection of individual privacy. However, the sanctions and the means of reprisal with regard to non-European companies that sell their products over the Internet are difficult to implement, which gives them relative impunity.

An approach, called Privacy by Design is gaining greater and greater importance. It includes the respect of privacy even earlier in the project, as early as the design stage, by ensuring the pertinence of the data collected and by anticipating the information provided to and the rights of access of users [16].

However, CHD providers must be conscious that technological development in re-identification of de-identified patient data has become a major policy concern. As reported by Lee Tien [17], only in the past few years we have begun to realize how difficult it is to truly de-identify data, given the enormous amount of information about people that is publicly available to data-miners, including hospital discharge summary databases. Modern re-identification techniques do not depend on personally identifiable information - any information that distinguishes one person from another can be useful. Researchers recently used modern techniques to re-identify supposedly anonymized genetic samples - determining not just the names of some of the people who submitted the sample in the first place but also their entire families.

It is also important that CHD providers anticipate in their technical development the required counter-measures to the main threats, which can jeopardise data integrity and therefore patients’ health. As expressed by Ohno-Machado [18], a particular attention has to be paid to replay attack and External Device Mis-Bonding. In a replay-attack, a valid data transmission could be maliciously repeated by a hacker. For example, an attacker can first record the communication from the sensor that indicates a high glucose level based on some auxiliary knowledge about the victim. Then, the attacker could later retransmit the high glucose information pretending it is a “valid” message, which would cause the receiver (e.g., insulin pump) to deliver an incorrect insulin dose and put the patient at risk. One way to avoid replay attacks is to introduce timestamps [19] within the message, where one CHD with healthcare applications only accepts the message from the other device if the timestamp in the received message is within a reasonable time tolerance range. In this case, a replay attack would not be able to provide a valid timestamp by simply reusing the previously sniffed transmission. However, time stamping requires synchronization with mHealth devices, which may impose additional communication burden and reduces battery life. Concerning DMB attack, two system security issues have to be taken into account: the information leakage risk and the information injection risk. For the first type, a malicious “app” on an authorized phone can steal sensitive patient data that are intended to be transmitted to an authorized app. In terms of information injection risk, an authorized app can feed false medical information into the original authorized app by intercepting the connection between the authorized external device and the authorized smartphone. This injection risk is extremely dangerous for patients who are heavily relying on health monitoring apps (e.g., blood-sugar concentration, irregular heart rhythm, etc.).

## The solutions: a posteriori control and the establishment of standards

Today, what differentiates the evaluation of usual medical devices from that of an application or a CHD is essentially that many of the latter are likely to include recordings of personal data; in addition, these devices will be used by large numbers of people, which gives the societal dimension and is likely to compromise individual privacy. Companies accept the legitimacy of the protections and guarantees required, but criticize the excessively long delays, which, as well as distorting competition, as already pointed out, will constitute a major intrinsic handicap for products whose lifespan is often very short. Any delay of a few months may render the device obsolete even before it has been authorized.

One of the proposals made on the occasion of the aforementioned symposium devoted to the technological and ethical evaluation of these healthcare applications, was to allow prior auto-certification by the manufacturer associated with an a posteriori control procedure to assess the measures implemented in the devices to protect individual privacy.

This auto-certification and a posteriori control process would constitute a guarantee while preserving the rapidity of authorization; it would require two essential elements: a standard and control measures.

The standard is fundamental in that each CHD manufacturer must have full knowledge of what commitments he has to make. The good or poor behaviour of the manufacturer will be measured against this standard, which will be established by all of the actors in the field, by the enterprises, of course, but in collaboration with healthcare professionals and associations of users of the healthcare system. A focus group will be set up at the national level to lay the foundations for this work by drafting recommendations. These will constitute the minimal prerequisites that must be respected with regard to data processing by the CHD with healthcare applications. The recommendations will then be submitted to the National Medical Council, and the national commission for data protection (CNIL) in France for approval. Contact will be made with European institutions to envisage their extension to different countries in the European Union.

With regard to control measures and sanctions, they have already been provided for in the French law relative to the protection of personal data in the context of missions assigned to the CNIL, which is an administrative authority. In terms of personnel, the human resources of the CNIL are already insufficient to handle all of the tasks assigned to it, and the exponential development of CHD may overwhelm the departments that deliver the authorizations. The consequence would be an increase in delays, the proportion of which would become completely intolerable. This is why it seems advantageous to promote a posteriori controls, which would require far fewer resources. It could be hoped that fear of the “policeman” will be sufficiently effective, on condition that a certain number of controls are indeed done. A posteriori control, if it is real, often encourages interested parties to take more precautions than is the case with a priori controls: if there is a problem, it is the whole structure and the totality of their investments that will collapse, without the possibility of negotiation and with the risk of heavy fines and even imprisonment.

## Conclusion

The market for Connected Health Devices (CHD) with healthcare applications is growing fast and should be worth several billion euros in turnover in the coming years. Their development will completely transform the organisation of European healthcare systems, change the way patients are managed and revolutionize disease prevention.. The new organisation should facilitate the life of patients as well as of doctors. However, the limits of such a system need to be explored, and the major question as to who will control these information systems should be discussed.

Many issues regarding the technological and medical validation of CHD with healthcare applications have to be addressed. These include the assessment of their value as a veritable healthcare service and guarantees that individual privacy is respected with regard to the information collected and processed by these devices. The societal consequences of CHD with healthcare applications have to be assessed. The need for a medical as well as ethical evaluation, and for security guidelines, which producers of connected devices could use to ensure compliance. Rather than trying to stop this tidal wave, we propose the implementation of a posteriori rather than a priori checks so as not to hamper innovation.

## Abbreviations

CHD: Connected Health Devices
CNIL: National commission for data protection
